# Impact of Rab27 on Melanoma Cell Invasion and sEV Secretion

**DOI:** 10.3390/ijms252212433

**Published:** 2024-11-19

**Authors:** Katarzyna Horodecka, Liliana Czernek, Łukasz Pęczek, Mariusz Gadzinowski, Magdalena Klink

**Affiliations:** 1 Centre of Molecular and Macromolecular Studies, Polish Academy of Sciences, 90-363 Lodz, Poland; katarzyna.horodecka@cbmm.lodz.pl (K.H.); liliana.czernek@cbmm.lodz.pl (L.C.); lukasz.peczek@cbmm.lodz.pl (Ł.P.); mariusz.gadzinowski@cbmm.lodz.pl (M.G.); 2 Institute of Medical Biology, Polish Academy of Sciences, 93-232 Lodz, Poland

**Keywords:** melanoma cells, Rab27, small extracellular vesicles, invasiveness

## Abstract

The migratory and invasive capabilities of melanoma cells contribute to metastasis. Therefore, targeting the genes driving these processes can support melanoma therapy. Rab27A and Rab27B contribute to tumor formation progression in many types of cancer through various mechanisms, including the secretion of small extracellular vesicles (sEVs). We explored the role of these GTPases in melanoma cell functioning in three RAB27A knockout (KO) cell lines (A375, DMBC12, and SkMel28) and a double RAB27A/B KO A375 cell line. The loss of RAB27A impaired the migration and invasion of DMBC12 and SkMel28 cells; however, the behavior of highly aggressive A375 cells was unaffected. The RAB27A/B double knockout moderately decreased the migratory capacity of A375 cells without disturbing their invasiveness. Additionally, the silencing of RAB27A did not affect the number and mean size of the sEVs, despite some alterations in the protein content of the vesicles. Both Rab27 isoforms can, at least partially, act independently. The potential role of Rab27A in the functioning of melanoma cells depends on the individual character of the cell line, but not on its basal expression, and seems to be unrelated to the secretion of sEVs.

## 1. Introduction

Malignant transformation of melanocytes leads to the formation of melanoma, the most aggressive type of skin cancer. While early-stage melanoma can often be effectively treated with surgery, advanced disease poses significant challenges due to the rapid invasion and metastasis, reducing the 5-year survival rate to 30% [1]. Therefore, exploring novel therapeutic strategies is still fundamental for improving long-term outcomes in advanced melanoma patients.

The identification of driver and passenger mutations revealed many potential targets for precise cancer therapy, including Rab27, a protein associated with progression and metastasis across various types of cancer [2,3,4]. Rab27A mRNA and protein were overexpressed in melanoma samples compared to normal skin or benign nevi. Furthermore, higher levels of Rab27A mRNA were correlated with worse survival outcomes in patients with stage III melanoma [5].

Rab27 belongs to a family of small GTPases and consists of two isoforms, Rab27A and Rab27B, sharing 71% amino acid similarity. They act as molecular switches, oscillating between active GTP-bound and inactive GDP-bound states. An active Rab GTPase interacts with effector proteins [6]. Rab27A regulates the transport of melanosomes in melanocytes by interacting with melanophilin, which participates in actin-based trafficking, and synaptotagmin-like protein 2-a, while docking melanosomes to the plasma membrane [7,8]. Melanosome transport is additionally coordinated by MITF, which regulates Rab27A expression by binding to its promoter [9]. Taking into account the oncogenic role of MITF in malignant melanocytes, this sheds light on the involvement of Rab27 in melanoma.

Rab27 was found to regulate the proliferation, migration, invasion, or clone formation of various types of cancer cells [10,11,12,13,14]. It is also thought to participate in the secretion of soluble factors, including metalloproteinases, cytokines, chemokines [15], and proangiogenic factors [16] by tumor cells. However, one of the most acknowledged functions of this small GTPase is its involvement in the secretion of a subtype of small extracellular vesicles (sEVs), originally defined as exosomes. Exosomes are endosomal-originating membranous vesicles that range from ~30 to 200 nm in diameter. Another subtype of extracellular vesicles—ectosomes/microvesicles (MVs)—is produced by the budding of the plasma membrane and has a diameter of around 50–1000 nm [17,18,19]. However, given that the distinction between extracellular vesicles is still not yet fully established, the latest guidelines suggest use of the term “sEVs” while referring to exosomes [20]. For this reason, we opted to use this term even when referring to other studies that originally used different nomenclature.

Small extracellular vesicles act as mediators of intercellular communication by transporting nucleic acids, metabolites, amino acids, proteins, and lipids. Their size, cargo, and function can differ depending on their origin, and even between sEVs derived from the same cell, which provides various affinities and impacts on recipient cells. They are involved in various physiological and pathological processes, including the immune response, embryonic development, regeneration, cardiovascular and neurodegenerative diseases, or cancer [17,18,19]. sEV-mediated crosstalk between cancer cells or between cancer cells and the tumor microenvironment delivers signaling molecules that induce tumor development, invasion, metastasis, and drug resistance [21,22].

Previously, Rab27A was found to control the secretion of sEVs in mouse B16-F10 and human SkMel28 melanoma cells and to promote primary tumor growth in mice [16]. On the other hand, another study reported that sEV secretion by human metastatic (WM164 and WM983C) and mouse (B16-F10) melanoma cells was independent of Rab27A [5]. Furthermore, Rab27A was thought to promote the motility [23] and proliferation [24] of various melanoma cell lines. Knockdown of Rab27A or Rab27B reduced tumor growth and metastasis in murine models of melanoma [16]. Regarding the importance of Rab27 in melanoma behavior, it seems reasonable to increase the number of studies focusing on Rab27 knockout as a melanoma treatment approach. The potential benefit of targeting Rab27 for melanoma therapy still requires more studies, as to date there is no clear and unequivocal explanation of its implications in the biological activity of melanoma cells. This study aimed to explore the role of Rab27A in the proliferation, migration, and invasion of various human melanoma cell lines, as well as its contribution to the secretion of small extracellular vesicles. We used two commercially available primary melanoma cell lines that differed in Rab27 expression and invasiveness potential and a primary patient-derived melanoma cell line. Melanoma is highly heterogenous and this diversity is reflected in melanoma cell lines [25]. Studying a diverse panel of cell lines, derived from both primary and metastatic sites, is necessary to better understand the complex nature of this type of cancer. In addition to previously described studies focusing on cells derived from metastatic sites, our findings highlight the functional role of Rab27 in cell lines derived from primary tumors, suggesting its potential involvement in earlier stages of the disease also.

## 2. Results

### 2.1. Differential Basal Expression of Rab27A in Melanoma Cells

**Figure 1:**
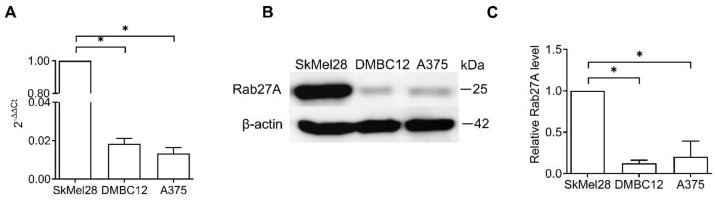
Melanoma cell lines show different levels of Rab27A mRNA and protein. The basal levels of RAB27A mRNA and Rab27A protein in SkMel28, DMBC12, and A375 melanoma cell lines were measured using qRT-PCR (**A**) and Western blot (**B**), respectively. (**A**) Data are presented as the mean ± SD of 3 independent experiments, * SkMel28 vs. DMBC12 or A375, *p* < 0.05 (one-way ANOVA with post hoc Tukey). (**B**) The representative Western blot of 3 independent experiments is shown. (**C**) The acquired bands were quantified by densitometric analysis and data are presented as the mean values ± SD of 5 independent experiments. * SkMel28 vs. DMBC12 or A375, *p* < 0.05 (one-way ANOVA with post hoc Tukey).

First, the expression of the Rab27A gene and protein was compared between melanoma cell lines using quantitative RT-PCR and Western blot, respectively. The highest level of RAB27A mRNA was observed in SkMel28 cells, while significantly lower levels were detected in A375 and DMBC12 cells (Figure 1A). The amounts of Rab27A protein were significantly higher in SkMel28 cells compared to the lower levels detected in A375 cells and DMBC12 cells (Figure 1B,C). We speculated whether the difference in the Rab27A levels translates to various functions of melanoma cells.

### 2.2. Loss of Rab27A Affects the Proliferative, Migratory, and Invasive Ability of Low-Invasive Melanoma Cells

**Figure 2:**
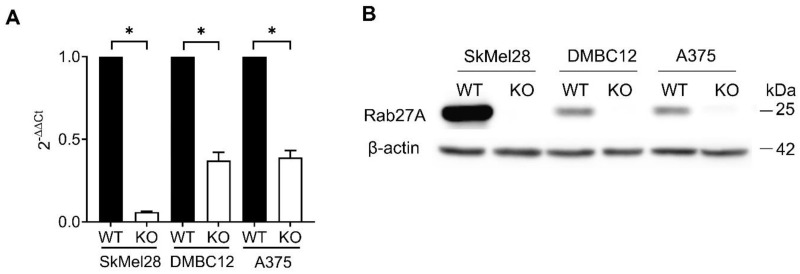
Confirmation of RAB27A knockout in SkMel28, DMBC12 and A375 melanoma cell lines. The RAB27A mRNA and Rab27A protein levels were detected using qRT-PCR and Western blot, respectively. (**A**) Data are presented as the mean ± SD of 3 independent experiments, * WT vs. KO, *p* < 0.05 (unpaired *t*-test). (**B**) The representative Western blot of 3 independent experiments is shown.

To investigate the function of Rab27A in melanoma cells, we used CRISPR/Cas9 technology to knock it out in A375, DMBC12, and SkMel28 cells. The downexpression of RAB27A mRNA was detected by RT-PCR (Figure 2A), while the absence of the protein was confirmed by Western blot (Figure 2B).

**Figure 3:**
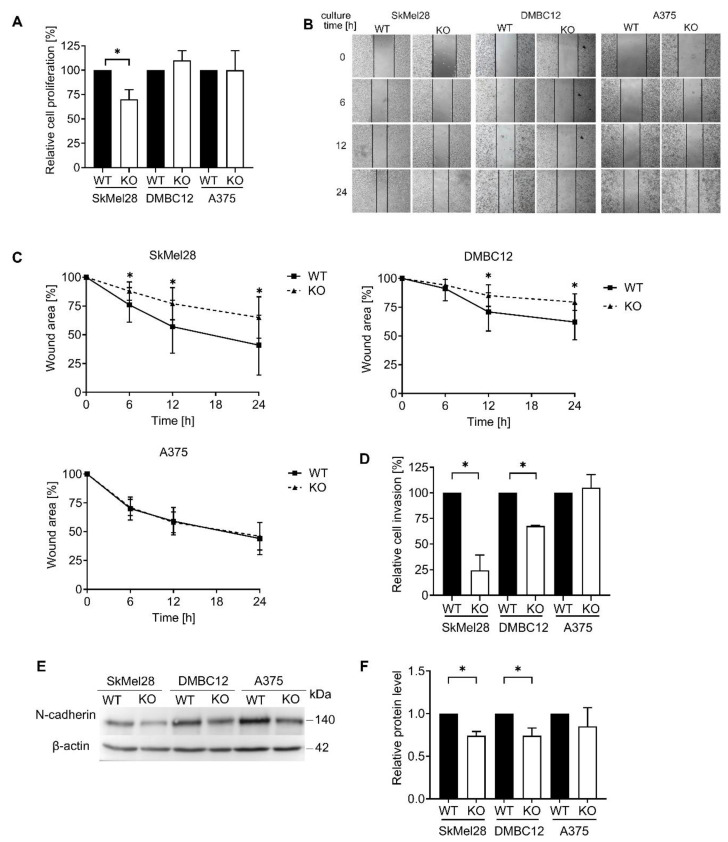
The impact of Rab27A on the proliferation, migration and invasion of melanoma cells. (**A**) Cell proliferation was assessed by fluorescent-based measurement of the DNA content. Data are presented as the mean values (±SD) of 3 independent experiments. * WT vs. KO, *p* < 0.05 (unpaired *t*-test). (**B**) Representative image of wound healing is shown. Magnification 40×. (**C**) The wound area was measured at different time points and normalized to the initial area at time 0. Data are presented as the mean values (±SD) of at least 4 independent experiments. * WT vs. KO, *p* < 0.05 (unpaired *t*-test). (**D**) Cell invasion was measured using chambers covered with a layer of extracellular matrix. The results are shown as the relative value of the invaded knockout cells compared to the control cells. Data are presented as the mean values (±SD) of at least 4 independent experiments. * WT vs. KO, *p* < 0.05 (unpaired *t*-test). (**E**) The protein levels of N-cadherin in WT and KO melanoma cell lysates were analyzed by Western blot assay. A representative image is shown. (**F**) The acquired bands were quantified by densitometric analysis and the data are presented as the mean values ± SD of at least 4 independent experiments. * WT vs. KO, *p* < 0.05 (unpaired *t*-test).

Cell proliferation, migration, and invasion are crucial properties of cancer cells that promote the expansion of surrounding tissues and eventually lead to tumor metastasis [26]. We studied cell proliferation by measuring the increment of the cellular DNA content. The proliferation of RAB27A knockout (KO) SkMel28 cells was inhibited compared to the wild type (WT). However, RAB27A KO DMBC12 and RAB27A KO A375 cells proliferated similarly to their respective controls (Figure 3A). We performed the wound-healing assay, which allows for measuring the two-dimensional cell migration. We observed a decrease in the migration of RAB27A KO DMBC12 and RAB27A KO SkMel28 cells compared to controls. Interestingly, the migration of RAB27A KO A375 cells was not impaired (Figure 3B,C). To gain further insight into the invasive phenotype of RAB27A KO melanoma cells, we analyzed invasion through the layer of extracellular matrix, which resembles the tumor microenvironment (Figure 3D). Similarly to the two-dimensional migration, we observed a decrease in the invasion of RAB27A KO DMBC12 and RAB27A KO SkMel28 cells compared to their corresponding controls. For A375 cells, the loss of Rab27A did not inhibit the cell invasion capability. Furthermore, we studied the levels of the mesenchymal marker, N-cadherin, which is known to directly affect melanoma cell invasion [27,28] (Figure 3E,F). Lower levels of N-cadherin were observed in RAB27A KO DMBC12 and RAB27A KO SkMel28 cells, which confirms that Rab27A controls the invasion of these cell lines.

In summary, Rab27A controlled the migration and invasion of low-invasive SkMel28 and DMBC12 cells, which corresponded to decreased levels of N-cadherin, while the behavior of the RAB27A KO A375 (highly invasive) cell line was not affected in any capacity. Only SkMel28 cell proliferation was found to be regulated by Rab27A.

### 2.3. Alterations of sEV Protein Content Induced by RAB27A Knockout

**Figure 4:**
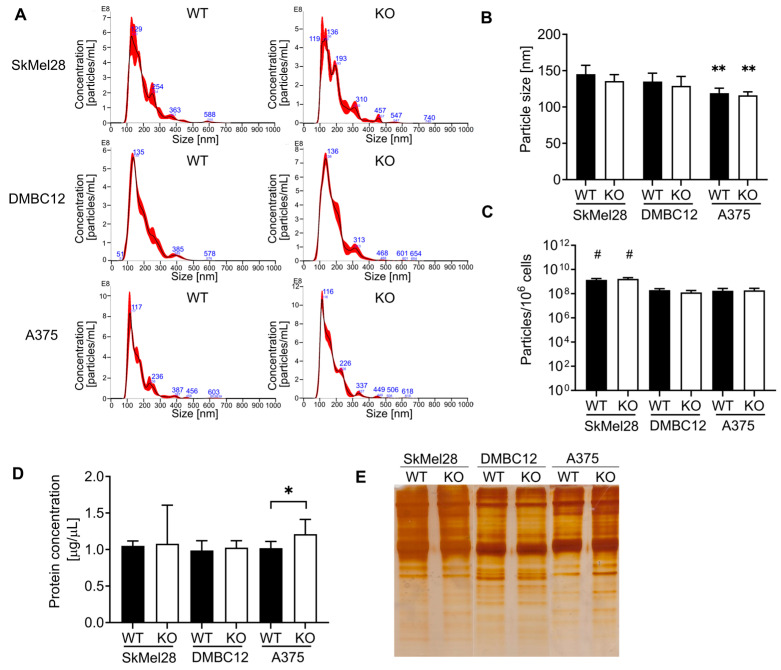
Characterization of sEVs secreted by WT and RAB27A knockout SkMel28, DMBC12, and A375 cells. (**A**–**C**) The size distribution and concentration of the secreted sEVs were analyzed by NTA. (**A**) Images of representative particle measurements are shown. The size (**B**) and concentration (**C**) of the particles are presented as the mean ± SD of at least 4 independent experiments, ** WT and KO A375 sEVs vs. WT and KO SkMel28 and DMBC12 sEVs, *p* < 0.05 # WT and KO SkMel28 sEVs vs. WT and KO DMBC12 and A375 sEVs, *p* < 0.05 (one-way ANOVA with post hoc Tukey). (**D**) The total protein concentration of the sEVs resuspended in equal volumes of PBS was measured by BCA assay. Data are presented as the mean ± SD of at least 4 independent experiments. * WT vs. KO, *p* < 0.05 (unpaired *t*-test) (**E**) The total protein content of the sEVs was also visualized by silver staining of SDS-PAGE. The representative images of 3 independent experiments are presented.

One of the crucial functions of Rab27 is the secretion of small extracellular vesicles. Therefore, we analyzed the number and size of the sEVs released by the RAB27A KO melanoma cells using nanoparticle tracking analysis (NTA) (Figure 4A–C). Depending on the cell line, their size ranged from ~119 to ~145 nm, which is in agreement with the characteristic sizes/dimensions of small extracellular vesicles. The mean particle size was comparable between WT and KO A375 sEVs and between WT and KO DMBC12 sEVs. Knockout cells appeared to release smaller particles than WT SkMel28; however, the difference did not reach statistical significance (*p* = 0.25). The mean size of the WT and KO A375-derived sEVs was significantly smaller in comparison to the WT and KO DMBC12- and SkMel28-sEVs (Figure 4A,B).

We observed that WT and KO SkMel28 cells produce more sEVs in comparison to WT and KO A375 and DMBC12 cells. The number of particles was similar between WT and RAB27A KO A375 sEVs and between WT and RAB27A KO SkMel28 sEVs. We observed fewer particles secreted by RAB27A KO DMBC12 cells compared to the wild type, although the difference was not statistically significant (*p* = 0.33) (Figure 4C). The size distribution of the vesicles released by KO cells exhibited nuanced variations compared to those released by WT (Supplementary Figure S1). SkMel28 RAB27A KO and DMBC12 RAB27A KO cells secreted more particles measuring 120–160 nm and 200–240 nm, and fewer particles measuring 160–200 nm, than their wild-type counterparts. A375 RAB27A KO cells released less sEVs with a 200–240 nm diameter. However, these fluctuations are very subtle and the differences between the abundance of each vesicle subpopulation do not exceed 8%. Moreover, the shift in particle size is not consistent across cell lines; therefore, it is probably not significant enough to affect cell functioning.

The total protein concentration of the sEVs secreted by WT and RAB27A KO SkMel27 and DMBC12 cells measured by the BCA assay was equal. However, the protein content of RAB27A KO A375 sEVs was higher than WT A375 sEVs (Figure 4D). Except for RAB27A KO A375 sEVs, the proportional protein content between the control and KO sEVs was also observed after separation by SDS-PAGE electrophoresis followed by silver staining (Figure 4E).

**Figure 5:**
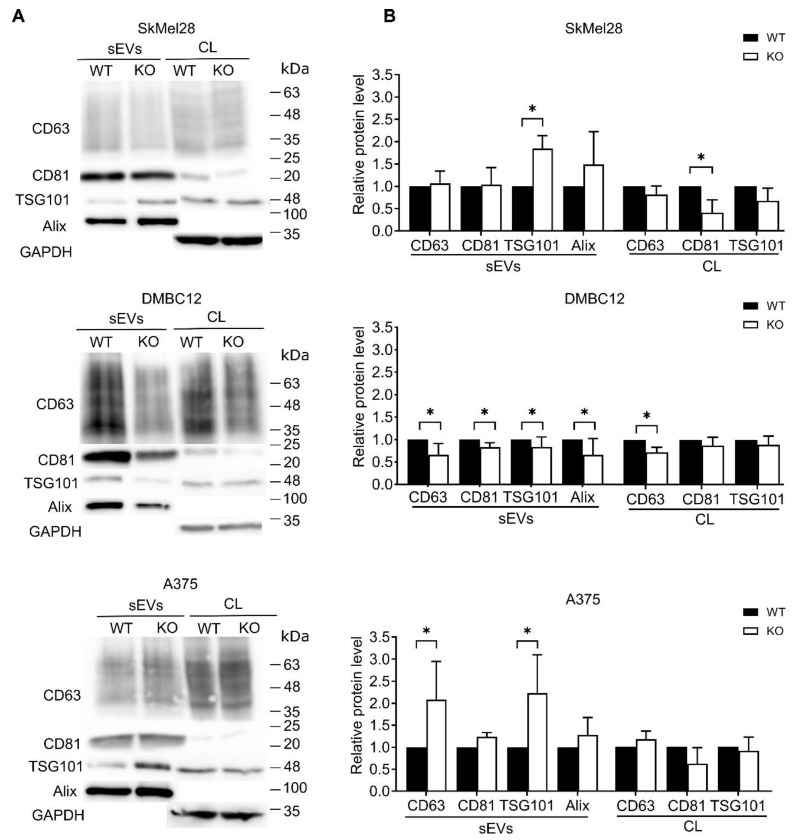
The protein levels of CD63, CD81, Alix, TSG101 in the sEVs and cell lysates (CLs) of WT and KO melanoma cell lines. An equal amount of protein from cell lysates or an equal volume of sEVs was analyzed by Western blot assay. (**A**) A representative image of the protein contents, along with GAPDH level, is presented. (**B**) The acquired bands were quantified by densitometric analysis and the data are presented as the mean values ± SD of at least 6 independent experiments, * WT vs. KO, *p* < 0.05 (unpaired *t*-test).

Next, we analyzed the expression of tetraspanins (CD63, CD81) and ESCRT-proteins (Alix and TSG101) in cell lysates and sEVs by Western blot (Figure 5A). The levels of CD63 and CD81 detected in wild-type and RAB27A KO SkMel28 sEVs were similar, while TSG101 was significantly elevated. The slight increase in Alix in RAB27A KO SkMel28 sEVs did not reach statistical significance (Figure 5B). The tetraspanin CD81 was down-regulated in RAB27A KO SkMel28 cells, while the remaining markers were at comparable levels in cell lysates. In contrast, we observed significantly decreased levels of CD63, CD81, Alix, and TSG101 in sEVs secreted by RAB27A KO DMBC12 cells in comparison to WT-sEVs. A lower amount of CD63 was detected in RAB27A KO DMBC12 cells compared to WT DMBC12 cells, while the other markers remained at similar levels. A significant increase in CD63 and TSG101 was seen in RAB27A KO A375 sEVs, but the other markers remained intact in comparison to the sEVs of WT A375 cells. The cellular levels of CD63, CD81, and TSG101 were similar in RAB27A KO and WT A375 cells. The barely detectable level of Alix in the cell lysates of all the tested cell lines did not allow for proper analysis.

Overall, these results indicate that Rab27A does not directly control the number of sEVs secreted by A375, DMBC12, and SkMel28 cells, regardless of the basal mRNA and protein levels. However, RAB27A KO changed the protein content of the sEVs differently depending on the cell line.

### 2.4. The Loss of Rab27A and Rab27B Does Not Affect the Number of Secreted sEVs and the Invasiveness of Highly Invasive Melanoma Cells

**Figure 6:**
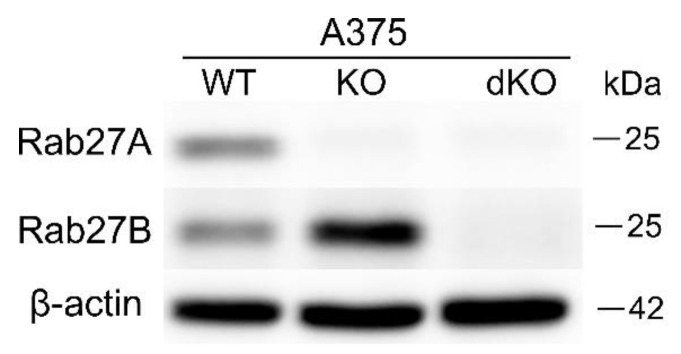
Confirmation of RAB27A and RAB27B knockout in the dKO A375 melanoma cell line using Western blot.

We observed the unanticipated effects of RAB27A knockout in A375 cells, which led to the hypothesis that another Rab27 isoform substitutes the function of Rab27A. We observed an increase in Rab27B in RAB27A KO A375 cells (Figure 6). Therefore, to rule out the possible compensation of Rab27A’s absence by Rab27B, we created a double knockout (dKO) RAB27A/B KO A375 cell line. Downregulation of Rab27A and Rab27B was verified using Western blot (Figure 6).

**Figure 7:**
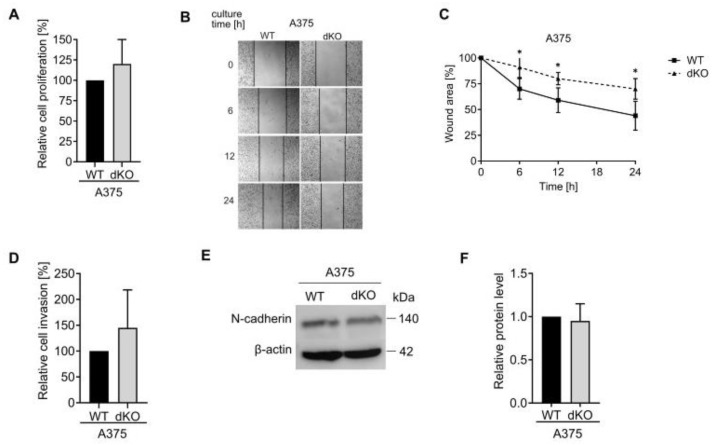
The impact of RAB27A and RAB27B double knockout on the proliferation, migration and invasion of A375 cells. (**A**) Cell proliferation was assessed by fluorescent-based measurement of the DNA content. Data are presented as the mean values (±SD) of 3 independent experiments. (**B**) Representative image of wound healing is shown. Magnification 40×. (**C**) The wound area was measured at different time points and normalized to the initial area at time 0. Data presented as the mean values ± SD of at least 3 independent experiments are shown, * WT vs. dKO, *p* < 0.05 (unpaired *t*-test). (**D**) Cell invasion was measured using chambers covered with a layer of extracellular matrix. The results are shown as the relative value of the invaded knockout cells compared to the control cells. Data are presented as the mean values ± SD of at least 4 independent experiments. (**E**) The protein level of N-cadherin in the WT and dKO A375 cell lysates was analyzed by Western blot assay. A representative image is shown. (**F**) The acquired bands were quantified by densitometric analysis and the data are presented as the mean values ± SD of 4 independent experiments.

The proliferation of RAB27A/B knockout cells was not suppressed (Figure 7A); however, we observed the inhibition of cell migration when analyzed by wound-healing assay, suggesting that Rab27B plays a more substantial role in this cell line motility (Figure 7B,C). Interestingly, the A375 cell invasion through the extracellular matrix was not suppressed by the double Rab27 knockout (Figure 7D). The level of N-cadherin did not change significantly, which is in line with the result of the invasion assay (Figure 7E,F).

**Figure 8:**
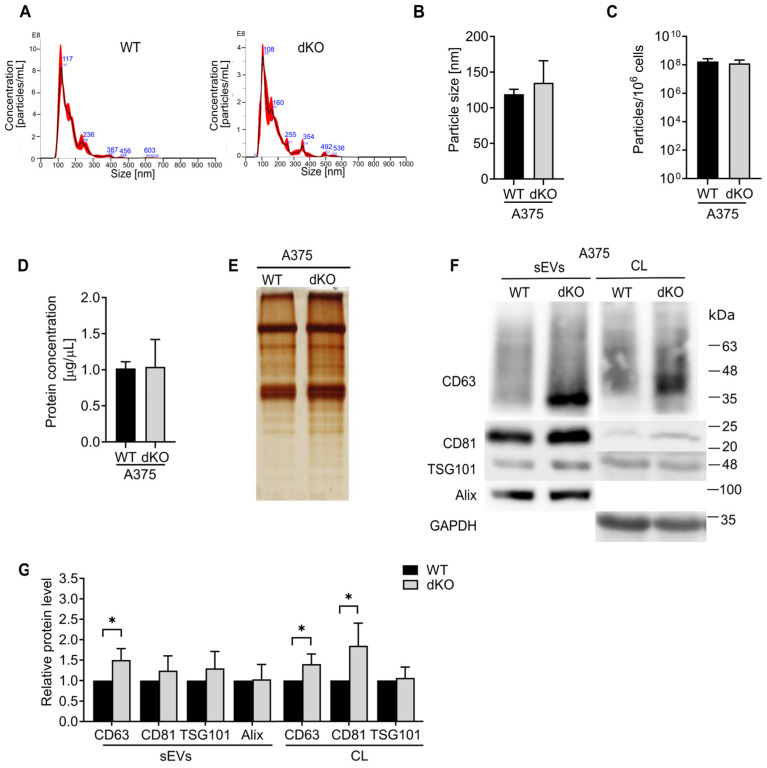
Characterization of sEVs secreted by WT and RAB27A/B double knockout (dKO) A375 cells. The size distribution and concentration of the sEVs were analyzed by NTA. (**A**) Images of the representative particle measurements are shown. The size (**B**) and concentration (**C**) of the particles are presented as the mean ± SD of at least 3 independent experiments. (**D**) The total protein concentration of sEVs resuspended in equal volumes of PBS was measured by BCA assay. Data are presented as the mean ± SD of 3 independent experiments, * WT vs. dKO, *p* < 0.05 (unpaired *t*-test). (**E**) The total protein content of the sEVs was also visualized by silver staining of SDS-PAGE. The representative images of 3 independent experiments are presented. (**F**) An equal amount of protein from cell lysates or an equal volume of sEVs was analyzed by Western blot assay. A representative image is presented. (**G**) The acquired bands were quantified by densitometric analysis and the data are presented the as mean ± SD of at least 6 independent experiments, * WT vs. dKO, *p* < 0.05 (unpaired *t*-test).

As previously discussed, the sEVs secreted by wild-type and RAB27A/B KO A375 cells were characterized. The size distribution and particle concentration measured by NTA were comparable (Figure 8A–C). No changes in the particle size distribution and concentration across different size ranges were observed (Supplementary Figure S1). A higher protein content of sEVs secreted by dKO A375 cells, compared to WT sEVs, was detected using BCA and silver staining assays (Figure 8D,E). Next, we studied the levels of CD63 and CD81, Alix, and TSG101 in RAB27A/B KO cells and sEVs. As shown in Figure 7F,G, similarly to RAB27 KO A375 sEVs, the level of CD63 was elevated in dKO sEVs compared to vesicles from WT cells. Contrary to RAB27A KO A375 sEVs, the TSG101 level in dKO sEVs was not elevated compared to wild-type sEVs. We also observed an increase in cellular CD81 in RAB27A/B KO cells compared to WT cells. The amount of other markers in the cell lysates and sEVs remained unaffected. All the above data indicate that the loss of both Rab27 isoforms appeared to be insignificant for the secretion of small extracellular vesicles.

These results provide further evidence that Rab27A and Rab27B do not affect the number of sEVs secreted by the A375 cells but significantly change their protein content. In addition, the knockout of both isoforms hinders cell migration but disturbs neither proliferation nor invasion of the A375 cell line.

## 3. Discussion

The importance of Rab27 proteins in the functioning of various types of cancer cells was reported in vitro and in vivo [10,11,12,29,30,31,32,33]. Therefore, we speculated whether the loss of RAB27A or RAB27A/B affected melanoma cell functioning. We found that the knockout of RAB27A in the DMBC12 and SkMel28 cell lines decreased their migratory and invasion potential. This indicates that either low or high Rab27A basal expression can play a significant role in these cells’ movement and aggressiveness, even though the involvement of Rab27A in the migration and spheroid invasion of melanoma cells has been reported to only occur when its basal expression is significant [5]. The inhibition of proliferation was observed in only RAB27A KO SkMel28 cells. Our reasoning is that the least invasive cell line was more susceptible to the weakening of its aggressive capabilities. The collected data are consistent with Guo et al. [5], who showed that RAB27A knockout in B16-F10 melanoma cells resulted in the impairment of cell motility. However, RAB27A KO A375 cells appear to migrate and invade extracellular matrix proteins similarly to WT cells. Double knockout in A375 cells inhibited migration, but the invasion capacity remained unchanged. The A375 cell line is characterized by an increased movement ability and greater invasiveness than the SkMel28 cell line. Furthermore, A375 cells release a higher amount of pro-invasive factors such as VEGF, MMP-2, or TNF-α (all necessary for the degradation of ECM) compared to less aggressive SkMel28 cells [34].

Rab27A’s involvement in invasion was previously described as related to the secretion of small extracellular vesicles [35]. Therefore, we decided to investigate whether the knockout of RAB27A or RAB27A/B disturbed the release of sEVs. Our study (based on the NTA and total protein content measurements) demonstrated that CRISPR-mediated knockout of RAB27A in various human melanoma cell lines (A375, DMBC12, and SkMel28) did not decrease the concentration of secreted sEVs or their total protein content. Moreover, the high basal level of Rab27A observed in SkMel28 cells did not influence its commitment to sEV release. Furthermore, double RAB27A and RAB27B KO in the A375 cell line did not lead to the inhibition of the number of sEVs released into the culture medium; thus, Rab27B did not compensate for the loss of Rab27A in these melanoma cells, even though Rab27B expression was enhanced significantly in RAB27A KO. These results are in line with those obtained by Guo et al. [5], who observed that the shRNA knockdown of RAB27A in WM164 and WM983C human melanoma cells did not affect the total number and protein content of secreted sEVs. Nevertheless, contrary to our studies, the shRNA-mediated RAB27A knockdown in SkMel28 cells resulted in a 50% decrease in the sEVs’ total protein content [15]. However, it should be noted that studying sEVs is very intricate and the results should be approached with caution. Discrepancies in the evidence on the Rab27A requirement in sEV generation from melanoma cells are unlikely due to the methodology used to silence it. Guo et al. [5] showed that shRNA- and siRNA-mediated knockdown, as well as CRISPR knockout of RAB27 in B16-F10 mouse melanoma cells, resulted in a comparable effect on the secretion of sEVs. Quantification of the mean particle size as well as analysis of the distribution across different size ranges revealed no meaningful differences in KO and WT cell lines. Nevertheless, the mean size of the sEVs originating from WT and KO of A375 cells was significantly smaller than those generated from both SkMel28 and DMBC12 cells. It was found by others that shRNA-mediated RAB27A knockdown in metastatic WM164 melanoma cells increased the population of smaller sEVs (<100 nm) [5]. The contrast in our and Guo et al.’s [5] results may be related to the different nature of the used cell lines.

Based on our findings, we assume that Rab27A is not a universal regulator in the vesicular trafficking in all kinds of malignant cells. It should be noted that other Rab GTPases, such as Rab3A, Rab7, Rab11, Rab35, and Rab37, are also important players in sEV biogenesis as they are involved in the requirement/docking of MVEs to the plasma membrane [36,37,38]. Especially, Rab3A may act complementarily with Rab27 since it shares some of the same effectors, such as rabphilin, which promotes dense-core-vesicle-docking to the plasma membrane [39]. Therefore, the participation of Rab proteins in sEV biogenesis could be complementary and/or cell-type-dependent [39]. The selective exclusion of one Rab can only slightly/partially alter the sEVs’ release pathway [40].

To examine whether the vesicular composition was altered due to the loss of Rab27, we decided to study the expression of the tetraspanins CD63 and CD81, and the ESCRT proteins ALIX and TSG101, as they are considered some of the most universal markers of sEVs [41,42]. The levels of CD63, CD81, and ALIX did not differ notably in the population of sEVs secreted by WT and RAB27A KO SkMel28 melanoma cells, while the level of TSG101 was enhanced in the sEVs of cells lacking Rab27A. On the other hand, we did not observe such an effect in the sEVs derived from RAB27A KO DMBC12 cells, which suggests that the relationship between TSG101, RAB27A and RAB27B is probably more complex, involving additional factors; therefore, further research is needed to fully understand this mechanism. Elevated levels of CD63 and TSG101 were also observed in the RAB27A KO A375 sEVs. This confirms that both proteins can be readily loaded into small extracellular vesicles through Rab27A-independent mechanisms [5]. Moreover, the high expression of Rab27A in SkMel28 does not guarantee its irreplaceable role in vesicular protein loading. As previously reported, this cell line is characterized by elevated expression of other Rab GTPases involved in sEV biogenesis and loading, i.e., Rab7 [16]. Notably, the addition of RAB27B knockout to RAB27A KO A375 cells restricted the loading of TSG101, but not CD63, in sEVs. This implies that both isoforms can independently modify the contents of vesicular proteins. The constant TSG101 level in RAB27A/B KO sEVs, unlike its upregulation in RAB27A KO A375 sEVs, suggests a compensatory role of Rab27B, which is absent when both GTPases are silenced.

Interestingly, we found that the absence of Rab27A in DMBC12 cells lowered the accumulation of all the above proteins in sEVs. This suggests that Rab27A, despite its low basal level, is strongly implicated in the loading of selected proteins into sEVs in this particular cell line. However, the decrease in the amount of characteristic proteins in sEVs cannot indicate inhibition of their secretion, as the concentration and total protein content remained unchanged, even though previous studies concluded the absence of sEVs based solely on the marker proteins’ decreasing level [23,40,43]. Fluctuations in TSG101 and CD63 were also observed in sEVs secreted by the RAB27A knockdown WM164 human melanoma cell line and the RAB27A knockout B16-F10 mouse melanoma cell line; however, this occurred without altering their total number [5]. On the other hand, it is noteworthy that one cell line secretes various populations of sEVs of distinct sizes and nucleic acid and protein compositions, which was observed in the mouse melanoma cell line B16-F10 [44,45]. Moreover, the expression of CD9, CD63, and CD81 was reported to differ even a hundredfold among vesicles released by HEK293 cells [46]. Therefore, it is possible that some populations of sEVs were more affected by the RAB27A knockout than others. A proteomic analysis of the secreted sEVs would be beneficial for a better understanding of the possible alterations in their cargo.

Our findings reveal that RAB27 knockout affects the sEV-associated protein content rather than their total secretion. The potential engagement of Rab27A and Rab27B in the loading of characteristic protein markers in the sEVs of melanoma cells is not related to their basal expression but is strictly dependent on the particular cell line. We hypothesize that the individual melanoma cell line can produce a different population of sEVs, probably depending on the distinct pathways involved in their biogenesis. The tested melanoma cells differ substantially not only in the Rab27 expression but also in a variety of other features. For example, SkMel28 cells are characterized as MITF high and low aggressive, while A375 and DMBC12 cells show low MITF expression and are considered pro-invasive [25,34]. MITF is known to regulate the expression of late endosomal proteins. Studies conducted on mouse melanocytes (Melan-α cell line) revealed that stable overexpression or knockdown of MITF substantially affected the levels of CD81 and various vacuolar protein-sorting-associated protein loading in sEVs [47].

Since we did not find any influence of Rab27 loss on the number of secreted sEVs, we assume that it can impact the pro-invasive composition of cell-derived vesicles. It was reported that sEVs from the metastatic BL6-10 melanoma cell line transferred the invasiveness molecules to the poorly metastatic F1 cell line [48]. Others described melanoma cell sEVs containing various signaling molecules from the MAPK family, HSP70, Tyrp2, and several microRNAs or pro-angiogenic factors (IL-8, VEGF, MMP-2, EGFR) that regulate the proliferation, migration, and invasion capabilities of neighboring cells [49,50]. Proteomics analysis of various melanoma cell lines implied that sEVs released by more aggressive cells contain more abundant proteins implicated in cell migration and angiogenesis [51]. The modulation of the CD63 and TSG101 content in sEVs of melanoma cells, as observed by us, was also described as important in promoting the more or less pro-invasive character of secretory vesicles [5]. However, based on our data, we cannot find a simple relation between the type of tetraspanin or ESCRT protein loading in sEVs and the invasiveness of melanoma cells.

It should be underlined that Rab27 is also known to control the sEV-independent secretion of soluble prometastatic factors like RANTES, metalloproteinases, PDGF, and PGF [16,52]. Another Rab27 activity, not related to sEVs, relies on its impact on the intracellular signaling pathway. Depletion of RAB27B (shRNA knockdown) was observed to reduce the phosphorylation of pro-survival ERK1/2 proteins in the TF-1 erythroblast cell line [53]. The loss of RAB27B in the HCC cell line (hepatocellular carcinoma) inhibited the PI3K/AKT signaling pathway, leading to suppression of cell proliferation [54]. Thus, the involvement of Rab27 in the invasiveness of melanoma cells appears to be more complicated than simple induction of sEVs generation.

Our studies included three human melanoma cell lines, with various Rab27A and Rab27B expression levels, as well as their proliferative/invasive potential. SkMel28 cells are less aggressive and feature significantly higher Rab27A/B expression and are characterized as MITF-enriched, which correlates with a proliferative state [25,34]. A375 cells are acknowledged to be a particularly invasive and aggressive type of melanoma that is MITF-negative and AXL-enriched [25,34]. In addition to commercially available cell lines, we studied the role of Rab27 in patient-derived DMBC12 cells, which were also characterized as a low MITF phenotype [55]. Rab27A and Rab27B levels are much lower in A375 and DMBC12 cells, which correlates with the low MITF expression. However, despite the higher levels of Rab27A in SkMel28 cells, the effects induced by the knockout are comparable to those observed in DMBC12 cells. Our findings suggest that the involvement of Rab27A is cell-line-dependent, rather than resulting from abundant protein levels.

In summary, we have shown that the Rab27A protein in SkMel28 and DMBC12 melanoma cells controls the migratory and invasion capabilities; however, it does not affect the number and protein concentration of the secreted sEVs. Since the sEV-dependent invasive capability of melanoma cells was reported, we hypothesize that Rab27A promotes the pro-invasive character of extracellular vesicles. Additionally, we cannot exclude the sEV-independent involvement of Rab27A in the secretion of soluble factors facilitating cell invasion. In contrast, in extremely aggressive A375 melanoma cells, Rab27A does not participate in either sEV biogenesis, cellular motility, or passage through the ECM. The loss of both Rab27A and Rab27B resulted in impaired A375 motility, but the invasive character and the generation of sEVs remained unaffected. This indicates that both Rab27 isoforms can act independently, at least partially. Importantly, our studies demonstrate that Rab27 cannot be described as a universal or key regulator of small extracellular vesicle secretion in all kinds of tumor cells. Even in the same malignancy, like melanoma, its activity differed depending on the character of the tumor cell. Our findings indicate that Rab27 possesses a more significant impact on the functioning of less-invasive melanoma cells. Therefore, targeting Rab27 could support anti-cancer therapy in some cases of this disease. This study open up several exciting avenues for further research, including a more thorough analysis of the complex interplay between Rab27A and Rab27B, which we plan to study in the future. Moreover, exploring the targeting of Rab27 in combination with other proteins involved in sEV secretion, such as TSG101, could additionally improve the therapeutic outcome and become a novel strategy for preventing melanoma progression and metastasis.

## 4. Materials and Methods

### 4.1. Cell Culture

The A375 human melanoma cell line and a patient-derived DMBC12 melanoma cell line isolated from a primary tumor (described in ref. [55]) were a kind gift from Prof. Malgorzata Czyż, Medical University, Lodz, Poland. The A375 cell line was authenticated by Eurofins Genomics (Europe Applied Genomics GmbH, Ebersberg, Germany). The SkMel28 human melanoma cell line was purchased from the American Type Culture Collection (ATCC) (Manassas, VA, USA). All the cells were cultured at 37 °C with 5% CO_2_ in culture medium: RPMI 1640 medium (Corning, NY, USA) supplemented with 10% filtered fetal bovine serum (FBS) (EurX, Gdansk, Poland), 100 U/mL penicillin (Corning, NY, USA), 100 µg/mL streptomycin (Corning, NY, USA) and 2 mM L-glutamine (Corning, NY, USA).

### 4.2. Generation of CRISPR-Cas9 Knockout Cell Lines

The RAB27A and RAB27B knockout (KO) cell lines were created via transfection of ribonucleoprotein complexes containing Cas9 nuclease (Thermo Fisher Scientific, Waltham, MA, USA) and a single guide RNA (sgRNA) (Edit-R CRISPR (knockout) Human synthetic sgRNA) (Horizon Discovery, Lafayette, CO, USA). Three RAB27A-targeted sgRNAs (ATATTTCTCTGCGAGTGCTA, GTTCCATTCGCTTCATTATC, GCGTTCTTCAGAGATGCTAT), three sgRNAs targeting RAB27B (TGACTTCCCTCTGATCTGGT, TATAGTATTAATTGGCAACA, TTGCCAATTAATACTATATC), positive control (sgRNA targeting human PPIB gene—GGTGTATTTTGACCTACGAAT), and negative control (sgRNA not targeting human genome—CAAAACAGCATAGCTCTAAAAC) were resuspended according to the manufacturer’s protocol in 10 mM Tris-HCl buffer at pH 7.4 and stored in aliquots at −20 °C. For the delivery of the ribonucleoprotein complexes (RNP), the lipofectamine CRISPRMAX Cas9 transfection reagent (Thermo Fisher Scientific) was used according to the manufacturer’s instructions. Briefly, RNPs were prepared by mixing sgRNA with Cas9 nuclease in a 1:1 molar ratio in Opti-MEM medium (Gibco/Thermo Fisher Scientific) and adding Cas9 Plus reagent. Ribonucleoprotein was added to the transfection reagent, previously diluted in Opti-MEM, and incubated for 5–10 min at room temperature. The complexes were added to cells and cultured for 2–3 days at 37 °C. Single-cell clones were obtained by limiting dilution. Transfected cells were seeded in 96-well plates at a density of 0.5 cells per well. Single cells were expanded and analyzed for the loss of the Rab27A or Rab27B protein by Western blot. Sanger sequencing of the target site was performed as a secondary screening for knockout clones.

### 4.3. RNA Isolation and Quantitative Real-Time Reverse Transcriptase PCR (qRT-PCR)

The total RNA was isolated using TriPure Isolation Reagent (Roche Diagnostics GmbH, Mannheim, Germany) according to the manufacturer’s instructions. The RNA concentration was determined by spectrophotometric measurement on a NanoDrop^TM^ ND 1000 (Thermo Fisher Scientific) and stored at −20 °C until further analysis. Quantitative reverse transcription–polymerase chain reaction (qRT-PCR) was carried out using the LightCycler^®^ RNA Amplification Kit SYBR Green I (Roche Diagnostics GmbH) according to the manufacturer’s instructions. The primer sequences were designed with the Primer3 program available at https://www.ncbi.nlm.nih.gov/tools/primer-blast/, accessed on 1 September 2021. The following primers were used: RAB27A (forward, 5′-GTGCTGTGTGGAAACAAGAGT-3′; reverse, 5′-TTTGTCCCATTGGCAGCACT-3′), GAPDH (forward, 5′-CATCATCTCTGCCCCCTCTG-3′; reverse, 5′-TCCACGATACCAAAGTTGTC-3′). The isolated total RNA (250 ng/2.5 µL) and each primer (1 µL of a 2.5 mM solution) were added to the reaction mixture (5.5 µL) containing LC RT-PCR Reaction Mix SYBR Green I (2.0 µL), LC RT-PCR Enzyme Mix (0.2 µL), MgCl_2_ (5 mM, 0.8 µL) stock solution and PCR-grade water (LightCycler^®^ RNA Amplification Kit SYBR Green I, Roche Diagnostics GmbH). The amplification conditions were the following: reverse transcription of the RNA template at 55 °C for 10 min; deactivation of reverse transcriptase at 95 °C for 30 s; amplification of cDNA: denaturation (95 °C for 10 s), annealing (60 °C for 10 s) and extension (72 °C for 10 s). The products were identified by the thermal dissociation method and electrophoresis in 2% agarose gels. The cycle thresholds were normalized to the reference gene (GAPDH). The relative gene expression was calculated using the 2(−ΔΔCt) formula.

### 4.4. Isolation of Small Extracellular Vesicles

The culture medium was purified beforehand to remove extracellular vesicles derived from FBS. Firstly, FBS was filtered using a 0.22 μM pore filter membrane to dispose of larger vesicles before adding it to the culture medium. Secondly, the complete culture medium was ultracentrifuged at 100,000× *g* for 2.5 h at 4 °C to discard any remaining serum sEVs. The cells were cultured in purified serum sEV-depleted medium for 48 h prior to the sEV isolation, as previously described by our group [56]. The cells were counted using a Bürker chamber and seeded at equal density for each condition. During the medium collection, the cells were additionally counted to validate the proportionate cell growth, and their viability was confirmed. The culture medium from wild-type (WT), RAB27A knockout SkMel28, DMBC12, and A375 cells and RAB27A/B knockout A375 cells was collected and centrifuged at 300× *g* for 4 min to remove the detached cells. The supernatant was centrifuged for 30 min at 10,000× *g* at 10 °C to precipitate larger vesicles and cell debris. The supernatant was ultracentrifuged at 100,000× *g* for 2.5 h to pellet the extracellular vesicles. The pellet was washed in PBS and centrifuged again at the same speed. Each pellet was resuspended in an equal volume of filtered PBS. As a result, equal volumes of sEV solutions correspond to equal parts of the total amount of sEVs secreted by the same number of seeded cells. Portions of each resuspended sEV sample were used for different methods of analysis.

### 4.5. Nanoparticle Tracking Analysis (NTA)

Nanoparticle tracking analysis of the small extracellular vesicles was performed using the NanoSight NS3000 instrument (Malvern Instruments, Malvern, UK) with the NTA 3.4 Build 3.4.003 version. The sEV samples were diluted at 1:100 in filtered PBS. The capture settings were set to flow rate: 50, camera level: 12, threshold detection: 5, capture: 45 s, number of captures: 3, temperature: 25 °C, number of frames: 1124, 100–150 particles/frame.

### 4.6. Protein Quantification and Visualization

The total protein concentration of the cell lysates and sEVs was measured using a BCA Micro BCA™ Protein Assay Kit (Thermo Fisher Scientific) according to the manufacturer’s instructions. Silver staining of the SDS-polyacrylamide gels was performed to visualize the protein content of the sEVs and cell lysates. Here, 1× Laemmli sample buffer was added to the lysates or sEVs before boiling them for 5 min at 95 °C. Equal volumes of samples were separated by SDS-PAGE electrophoresis. The gels were fixed in 30% ethanol and 10% acetic acid for 1 h, followed by a 10 min rinse in 20% ethanol and a 10 min rinse in distilled water. The gels were treated with 0.2 mg/mL sodium thiosulfate for one minute and washed twice in distilled water. Staining with 10 mg/mL of silver nitrate was performed for 30 min, followed by washing twice with distilled water. The silver staining was developed using 30 mg/mL potassium carbonate and sodium thiosulfate (10 µg/mL) with 0.04% formaldehyde (37%) until intense bands occurred. The development was stopped in 10% ethanol and 5% acetic acid.

### 4.7. Western Blot

The cells were lysed with RIPA buffer (Sigma Aldrich, St. Louis, MO, USA) supplemented with protease inhibitor tablets (Roche Diagnostics GmbH) for 30 min on ice, followed by centrifugation for 15 min at 14,000× *g* at 4 °C. The supernatant was collected and stored at −20 °C until further use. Then, 1× Laemmli sample buffer was added to the lysates or sEVs before boiling them for 5 min at 95 °C. Non-reducing Laemmli buffer was used for the analysis of the CD63 or CD81 proteins. An equal amount of protein from cell lysates or an equal volume of sEVs resuspended in PBS was loaded onto 12% SDS-polyacrylamide gel. The proteins were separated by SDS-PAGE electrophoresis run for 1.5 h at 180 V, followed by a wet transfer to PVDF membrane for 90 min at 270 mA. The membranes were briefly washed in Tris-buffered saline with 0.05% Tween-20 (TBST) (Thermo Fisher Scientific, Waltham, MA, USA) and blocked using 5% BSA in Tris-buffered saline with 0.05% Tween-20 (TBST) (for 1 h at room temperature). The membranes were incubated with primary antibodies overnight at 4 °C. Subsequently, the membranes were washed with TBST three times and incubated with a horseradish peroxidase-conjugated secondary antibody for 1 h at room temperature. The membranes were washed with TBST. The ECL substrate (Clarity Western ECL Substrate, Bio-Rad, Hercules, CA, USA) was used to image the chemiluminescence of the membranes using the Gel Documentation System (Uvitec Ltd., Cambridge, UK). Densitometric analysis was performed using ImageJ software, version 1.53k.

The following primary antibodies were used: anti-Rab27a (D7Z9Q, Cell Signaling Technology, Danvers, MA, USA); anti-Rab27b (13412-1-AP, Proteintech, Manchester, UK); anti-CD63 (sc-5275, Santa Cruz Biotechnology, Dallas, TX, USA); anti-CD81 (M38, Invitrogen, Carlsbad, CA, USA): anti-Alix (sc-271975, Santa Cruz Biotechnology); anti-TSG101 (sc-7964, Santa Cruz Biotechnology); anti N-Cadherin (A19083, Abclonal, Woburn, MA, USA), anti-GAPDH (D16H11, Cell Signaling Technology); and anti-β-actin (13E5, Cell Signaling Technology). The following HRP-conjugated secondary antibodies were used: rabbit anti-mouse (P02060, Agilent Dako, Santa Clara, CA, USA) and goat anti-rabbit (P0448, Agilent Dako).

### 4.8. Proliferation Assay

Cell proliferation was measured using the CyQUANT™ Cell Proliferation Assay (Invitrogen) according to the manufacturer’s protocol. The cells were seeded in 96-well culture plates at a density of 1.5 × 10^3^ cells/well and grown in 150 μL of complete medium for 72 h. The culture medium was gently removed and 100 μL of 1× DNA-binding dye binding solution was added. The plates were incubated for 30 min at 37 °C. The fluorescence was measured at a wavelength of 480/520 nm on a microplate reader FLUOstar Omega (BMG Labtech, Offenburg, Germany). Blank values were subtracted. For each independent experiment, two to four wells per condition were measured. The mean fluorescence intensity of each condition was normalized to the mean fluorescence intensity of WT cells to calculate the relative proliferation rate.

### 4.9. Wound Healing Assay

All the tested cell lines were seeded into 24-well culture plates at a density of 1 × 10^5^ cells/well and grown in 1 mL of complete medium for 24 h. The confluent monolayer of the cells was scratched using a sterile P-20 pipette tip and the cells were washed twice with PBS. Culture medium supplemented with 1% FBS and antibiotics, without L-glutamine, was added and the cells were cultured for 48 h. Images were taken immediately after wounding and after 6, 12, and 24 h of culture using a camera attached to an Eclipse TS100 microscope (Nikon, Tokyo, Japan). For each independent experiment, four to eight wells per condition were analyzed. The wound areas were measured using ImageJ software (version 1.53k) with a wound-healing size tool [57]. The measurements were normalized to the wound area at the initial time point to calculate the wound closure.

### 4.10. Cell Invasion Assay

Cell invasion was studied using the QCM ECMatrix Cell Invasion Assay (Merck Millipore, Burlington, MA, USA) according to the manufacturer’s protocol. Beforehand, the cells were starved for 24 h in serum-free RPMI 1640 medium. Then, A375, RAB27A KO A375, RAB27A/B dKO A375 (0.35 × 10^6^ cells/mL), SkMel28, RAB27A KO SkMel28 (0.5 × 10^6^ cells/mL), DMBC12, and RAB27A KO DMBC12 (1 × 10^6^ cells/mL) cells were suspended in serum-free medium and added to the rehydrated invasion chambers coated with ECMatrix^TM^. A medium supplemented with 10% FBS (or serum-free medium, as a control) was added to the lower chamber. Cells migrate toward chemoattractant (FBS) and attach to the bottom of the chamber. After 48 h of incubation, the invaded cells were removed from the bottom and lysed with a lysis buffer containing fluorescent dye. The fluorescence was measured at a wavelength of 480/520 nm on a FLUOstar Omega microplate reader (BMG Labtech, Offenburg, Germany). Blank values were subtracted. For each independent experiment, two to four inserts per condition were used. The mean fluorescence intensity of each condition was normalized to the mean fluorescence intensity of WT cells to calculate the relative invasion rate.

### 4.11. Statistical Analysis

All the results were presented as the means, and as a measure of the dispersion from individual data values to the mean, the standard deviation (SD), was used. Statistical analysis was started by identifying outliers (Grubbs’ test) and assessing the normality of the given empirical distributions—Shapiro–Wilk W test. Subsequently, the homogeneity of the variances was verified by Levene’s test and the Brown–Forsythe test. Differences between two independent groups of data were calculated using the parametric test, Student’s *t*-test, or the nonparametric Mann–Whitney U test. To compare the means from three or more groups, the one-way ANOVA test (indicating an overall statistically significant difference in the group means) with the post hoc Tukey honest significant difference (HSD—was run to confirm where the differences occurred between groups) or nonparametric Kruskal–Wallis one-way analysis of variance by ranks followed by Dunn’s multiple comparisons test (to identify which groups are different) were used. All the statistical analyses were performed using Statistica ver. 8.0 software (StatSoft Inc., Tulsa, OK, USA). A value of *p* < 0.05 was considered statistically significant and was marked on the figures as *.

## Data Availability

The data that support the findings of this study are available from the corresponding author upon reasonable request.

## Supplementary Materials

The following supporting information can be downloaded at: https://www.mdpi.com/article/10.3390/ijms252212433/s1.
