# Supplementary material for: Impact of Rab27 on Melanoma Cell Invasion and sEV Secretion
Source: Int J Mol Sci. 2024 Nov 19;25(22):12433. doi: 10.3390/ijms252212433 (PMC11594641; doi:10.3390/ijms252212433)
Supplement: Supplementary file 1 [file ijms-25-12433-s001.zip › ijms-3289353-supplementary.pdf]

Impact of Rab27 on Melanoma Cell Invasion and sEVs Secretion

Katarzyna Horodecka, Liliana Czernek, Łukasz Pęczek, Mariusz Gadzinowski and Magdalena Klink

Supplementary

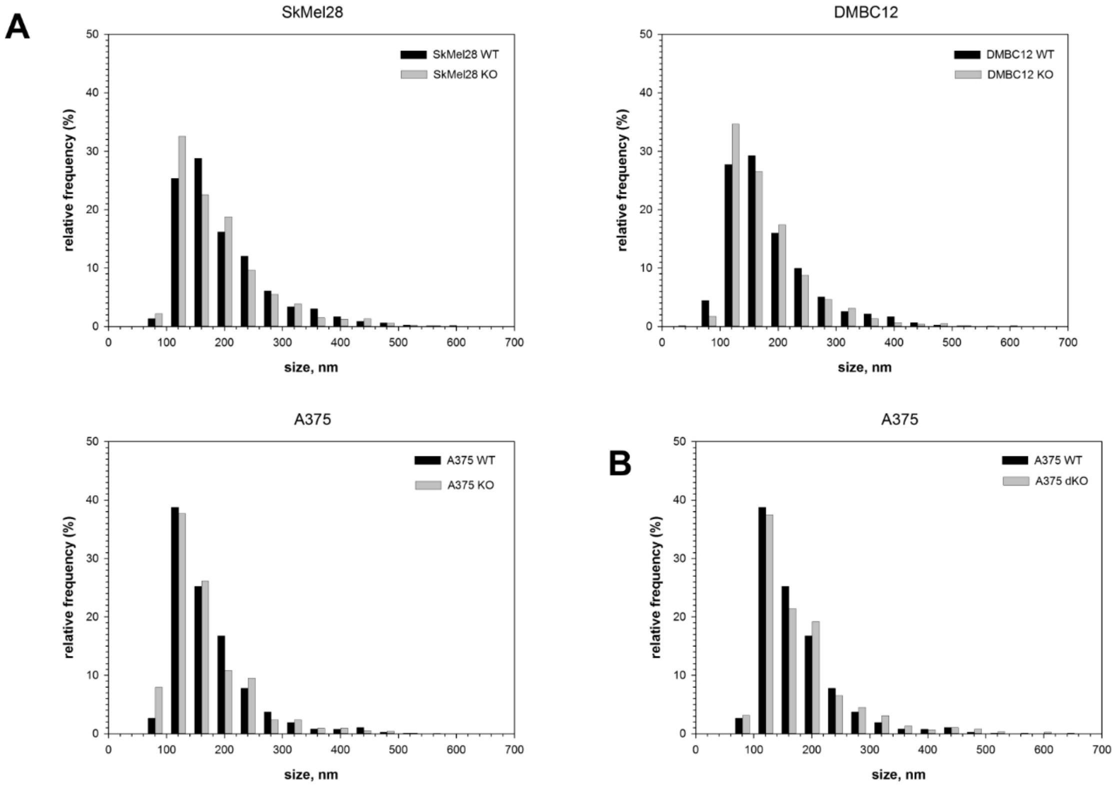

Supplementary Figure 1. Size distribution of sEVs secreted by wild-type (WT) and RAB27A knockout (KO) SkMel28, DMBC12 and A375 cells (A) and RAB27A/B A375 cells (B) analyzed by NTA.
